# Thermal Regulation of the Brain—An Anatomical and Physiological Review for Clinical Neuroscientists

**DOI:** 10.3389/fnins.2015.00528

**Published:** 2016-01-21

**Authors:** Huan Wang, Miri Kim, Kieran P. Normoyle, Daniel Llano

**Affiliations:** ^1^Department of Neurosurgery, Carle Foundation HospitalUrbana, IL, USA; ^2^Thermal Neuroscience Laboratory, Beckman Institute, University of Illinois at Urbana-ChampaignUrbana, IL, USA; ^3^University of Illinois College of Medicine at Urbana-ChampaignUrbana, IL, USA; ^4^Neuroscience Program and Department of Cell and Developmental Biology, University of Illinois at Urbana-ChampaignUrbana, IL, USA; ^5^Department of Molecular and Integrative Physiology, University of Illinois at Urbana-ChampaignUrbana, IL, USA; ^6^Department of Child Neurology, Massachusetts General HospitalBoston, MA, USA; ^7^NeuroTech Group, Beckman Institute, University of Illinois at Urbana-ChampaignUrbana, IL, USA; ^8^Department of Neurology, Carle Foundation HospitalUrbana, IL, USA

**Keywords:** neuroanatomy, cerebrovasculature, cerebral thermoregulation, carotid rete, cerebral circulation

## Abstract

Humans, like all mammals and birds, maintain a near constant core body temperature of 36–37.5°C over a broad range of environmental conditions and are thus referred to as endotherms. The evolution of the brain and its supporting structures in mammals and birds coincided with this development of endothermy. Despite the recognition that a more evolved and complicated brain with all of its temperature-dependent cerebral circuitry and neuronal processes would require more sophisticated thermal control mechanisms, the current understanding of brain temperature regulation remains limited. To optimize the development and maintenance of the brain in health and to accelerate its healing and restoration in illness, focused, and committed efforts are much needed to advance the fundamental understanding of brain temperature. To effectively study and examine brain temperature and its regulation, we must first understand relevant anatomical and physiological properties of thermoregulation in the head-neck regions.

## Introduction

Brain temperature, traditionally viewed simply as an *order* parameter that passively reflects a collective state of brain activity, may also act as a *control* parameter, a dynamic fluctuating variable capable of modulating brain activity and function (Wang et al., 2014). The human brain utilizes 20–25% of the energy budget, compared with primates (8–10%), and other mammals (3–5%; Leonard et al., 2007; Squire, 2012). As a metabolically demanding organ with intense heat production, the functional activity, and energy efficiency of the human brain is exquisitely sensitive to fluctuations in temperature (LaManna et al., 1980; Howarth et al., 2012; Wang et al., 2014). At the cellular level, ionic currents, membrane potential, input resistance, action potential, nerve conduction velocity, and synaptic transmission have all been shown to be affected by minute temperature variations (Wang et al., 2014). In the brain, the neuronal electronic signaling may have a per-neuron energy cost as high as 10 times that of other cells in the body. Therefore, at both the system and cellular levels, the brain's thermoregulatory capacity may underlie the physiological and anatomical constraints of the size and processing capacity of the human brain (Yu et al., 2014).

As endotherms, the human body is able to maintain near constant core body temperature (36–37.5°C) over a broad range of environmental temperatures. The lifetime mean of core body temperature for all mammals and birds is in the narrow range of 36–40°C (Jessen, 2001). Temperature is one of the most critical factors in determining not only the biochemical and metabolic processing rates, but also brain-to-body weight ratios (Yu et al., 2014). All endothermic animals are bound together by two unique features: larger brains and higher body temperatures; attributes that co-evolved and appear interdependent. Compared to primates of comparable size, the modern human brain is three times larger (Bruner, 2004) implying that the human brain more efficiently and effectively mitigates factors which would otherwise limit brain size and complexity, such as aberrant temperature. Recently, brain temperature has received intense clinical attention as an independent therapeutic target (Wang et al., 2014). One such appealing clinical application is the use of targeted brain cooling devices to manage head injuries in sports and trauma, especially notable in the case of mild TBI (Wang et al., 2015). Mild states of cerebral hyperthermia as induced by mild TBI, high ambient temperatures, or by physical activity and associated elevation of core body temperature affects several processes including the integrity of the blood brain barrier, mitochondrial function, and decreased tolerance to potential insults to the brain (as reviewed in Wang et al., 2015).

While the understanding of global hyperthermia is well established, the inability to measure brain temperatures with sufficient special and temporal resolution as well as regulation on a more quantal scale represent fundamental obstacles to developing effective therapeutic interventions. Effective targeted therapeutic interventions have increasing potential to minimize thermal and traumatic injury and may accelerate healing processes and functional restoration of the brain during chronic illness. In order to precisely develop tools to map the brain with high thermal resolution and effectively study brain temperature regulation, the anatomical and physiological properties in the head-neck regions must first be understood.

## Overview

Overall, core brain temperature is higher than core body temperature; however, these two temperatures correlate very well (Wang et al., 2014). Heat dissipation in the head is necessary due to continuous heat production (Yablonskiy et al., 2000), and tight control occurring via radiation, conduction, convection, and evaporation is equally necessary. The bony and soft tissue anatomical features covering the brain—meninges, skull, scalp and hair -have different thermal properties shielding the brain from thermal challenges, collectively maintaining temperature homeostasis and providing a means of buffering the superficial cortical regions from extreme temperature changes. When surgically exposed, the cerebral cortex may have temperatures that drop 5–10°C below core body temperatures (Gorbach et al., 2003; Kalmbach and Waters, 2012). This change is especially notable when a large piece of skull is removed, often performed to relieve intracranial pressure after brain injury, thus increasing the brain's thermal susceptibility to the external environment (Nakagawa et al., 2011; Suehiro et al., 2011).

**Figure 1:**
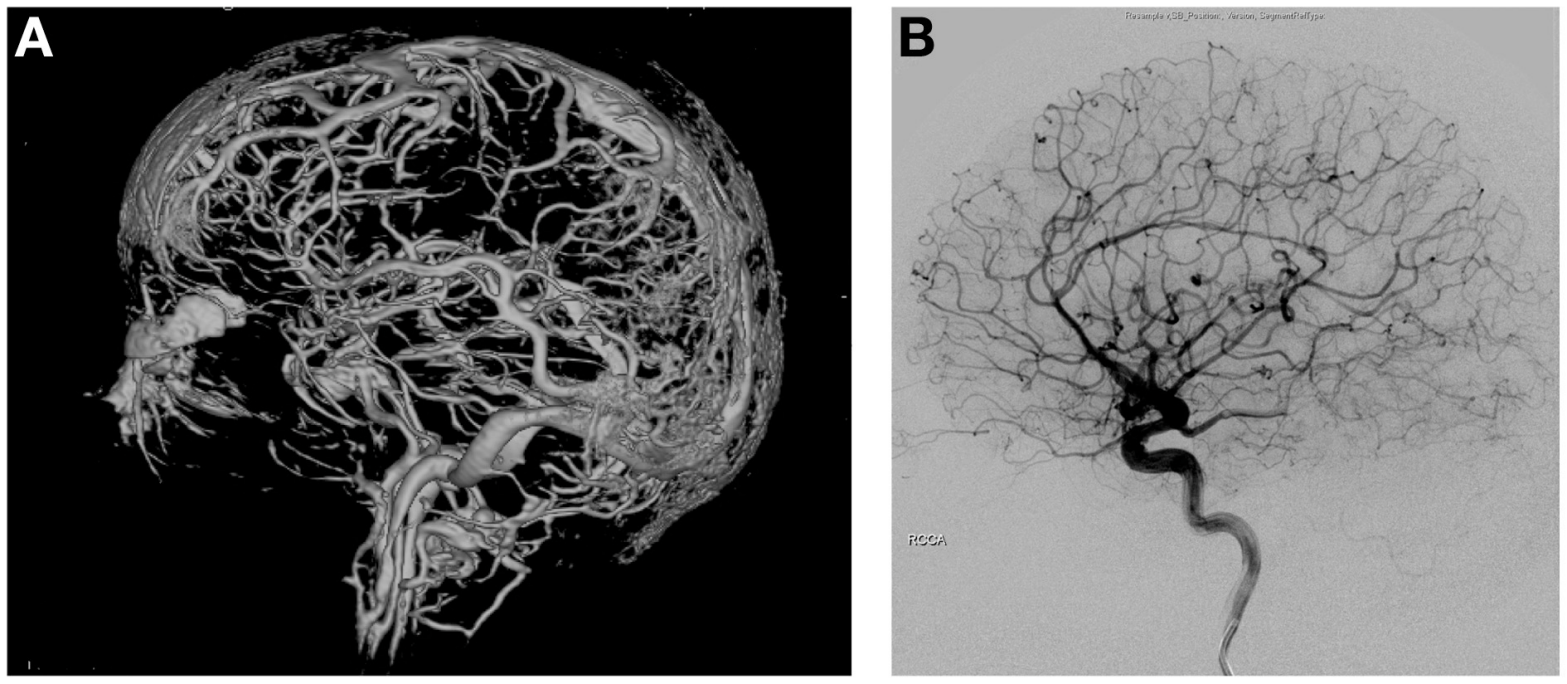
**(A)** 3D reconstruction of vascular networks in the brain. The intricate network of vessels of the arterial, venous, as well as scalp vessels can be appreciated creating a robust network of thermoregulation. **(B)** Lateral angiographic view of arterial phase of the brain.

The extraordinarily dense and robust vascular networks in the scalp, meninges, and brain (Zenker and Kubik, 1996) provide a highly effective thermal shielding mechanism against the environment, typically having a much lower average temperature than the brain temperature (Figures 1A,B). Without blood flow, the heat exchange properties in the head and neck regions rapidly change. In a monkey model of cardiac arrest at 35°C ambient temperature, it was noted that superficial sites on the brain cooled immediately while deeper brain temperatures initially increased. However, after 8–10 min, deeper intracranial sites cooled in parallel to surface temperatures (Hayward and Baker, 1969) demonstrating the brain's remarkable ability to rapidly adjust to thermal insults. Increasing the ambient air temperature to 45°C eliminated the pattern of temperature change observed at 35°C (Hayward and Baker, 1969). This illustrates the interrelationship between internal and external thermal influences and tissue- and organ-level responses of the brain. The scalp has also been demonstrated to influence the thermal autoregulatory apparatus of the brain. Humans diagnosed with brain death have significantly cooler scalps compared to core body temperature as previously described in children where a rectal-scalp temperature difference of greater than 4°C, which correlates with clinical criteria established for brain death (Miller et al., 1999).

## Cerebral blood flow (CBF)

CBF is most critical for maintaining and stabilizing the thermal environment of the brain. Under normal conditions, cerebral tissue beyond 2–3 cm from the cortical surface remains unaffected by changes in ambient temperature surface without simultaneous temperature changes in the perfusing blood (Stone et al., 1997). Core brain temperature is generally higher than body temperature; with blood temperature in the jugular vein higher than in the carotid artery (Nunneley and Nelson, 1994), CBF primarily contributes to heat removal from brain tissue. The superficial parts of the brain, however, are much more susceptible to the ambient temperature and may be cooler than arterial blood, particularly in neonates and infants (Iwata et al., 2014). Primate studies demonstrated that shifts in temperature, 5–7°C on either side of the neutral zone (28 and 32°C), did not affect deep brain structures, while superficial sites and CSF of the basal subarachnoid space were reported to change (Hayward and Baker, 1968). CBF, therefore, is critical in maintaining intracranial thermal homeostasis by reducing temperature in the deep brain but sometimes increasing temperature in the superficial brain (Iwata et al., 2014).

One such example of brain temperature regulation in mammalian species is the carotid rete. The carotid rete is a well-recognized mechanism for brain-specific cooling in rete species such as cats, dogs, (Daniel et al., 1953), and various artiodactyls (sheep, goats, pigs, etc.; Hayward and Baker, 1969). It is a compact plexus of intertwined, freely anastomosing arteries lying within a venous lake (Daniel et al., 1953). While not a human feature, the carotid rete is of great interest to those wishing to understand the mechanism of CBF contribution to brain temperature regulation. The venous lake around the plexus acts as a heat sink and facilitates substantial arterial heat dissipation to the venous blood before the arterial blood enters the Circle of Willis. Importantly, while the carotid rete represents an efficient counter-current mechanism of brain cooling, it is unclear how this mechanism could be robust to spatiotemporal thermal regulation within the brain as heat exchange is accomplished as blood enters/exits the organ. Understanding the carotid rete and thermal systems in non-rete species, will influence the translatability of animal model systems to humans.

**Figure 2:**
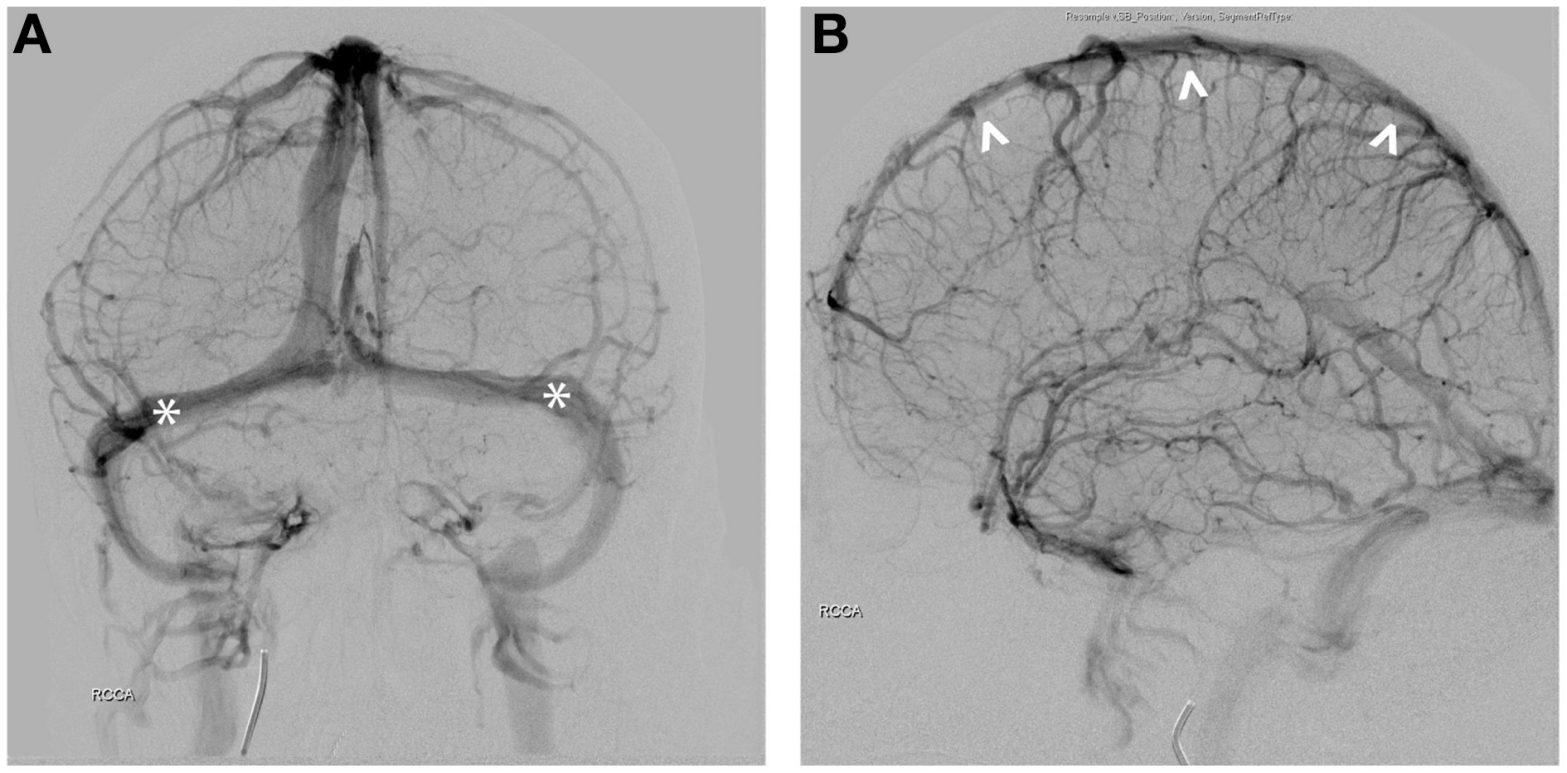
**(A)** AP view of venous phase. Seventy to Eighty percent of cerebral blood circulates in the venous vessels. Transverse sinuses are indicated (^*^). **(B)** Lateral view of the sinuses demonstrates close proximity of the superior sagittal sinus (^∧^) to the scalp.

The human brain contains many intimate contacts between arterial and venous flows in the cavernous sinus and vertebral venous plexus. However, these contact zones are short and the diameter of the traversing internal carotid and vertebral arteries are large, limiting thermal exchange, a problem further exacerbated by the relative thickness of the arterial wall and the rapid velocity of blood flow (Zenker and Kubik, 1996). In humans, the function of the carotid rete may be accomplished by arterial-CSF contacts. The cortical arterial branches traverse up to 20 cm within the subarachnoid space. Similar to the carotid rete, these vessels are thin-walled with similar vessel diameters (Simoens et al., 1987). Given the structural similarities to the carotid rete, these arterial vessels throughout the cortex are thought to have highly effective thermal interactions between the cerebral spinal fluid (CSF)-arterial blood flow (Zenker and Kubik, 1996). However, 70–80% of the cerebral blood volume circulates in the venous vasculature. Cortical veins, like the cortical arterial vessels, are thin-walled and also traverse long distances within the subarachnoid space (Figures 2A,B). The cortical veins circulate blood at low velocity and pressure compared to the arterial flow, likely providing a second means of highly effective CSF-venous thermal interaction in the brain, thus increasing the ability for thermal of regulation. The nature of having parallel high- and low-velocity systems may underlie both the robust nature of overall brain temperature regulation as well as the ability to spatiotemporally regulate temperature in various brain structures.

## Cerebrospinal fluid (CSF)

The CSF provides a second fluid circulation system for the CNS, analogous to the lymphatic system for the rest of the body (Cushing, 1914; Taketomo and Saito, 1965; Milhorat, 1975). The CSF around the brain acts as a suspensive force, reducing the effective weight of the brain from 1500 to only 50 g (Segal, 1993). With the intimate contacts with vasculature and brain parenchyma, the CSF is vital to the structural, biochemical, and thermal health of the brain (Wolfson et al., 1974; Segal, 1993; Zenker and Kubik, 1996; Redzic et al., 2005; Johanson et al., 2008).

CSF flow is intimately associated with the blood circulation from the interstitial, perivascular spaces (formally called Virchow-Robin spaces), through the capillary level and into the subarachnoid space (Hutchings and Weller, 1986; Esiri and Gay, 1990; Zhang et al., 1990). The contact surface area between the CSF and the brain is extraordinarily large, approximated to be 2300 cm^2^ in adult humans (Elias and Schwartz, 1969), with the fluid-brain contact surface at the capillary level through the perivascular and interstitial spaces estimated to be 250 cm^2^/g of tissue (Crone, 1963; Raichle, 1983). Past studies using tracers confirmed functional continuity within the subarachnoid, ventricular, perivascular, and interstitial compartments (Wagner et al., 1974; Rennels et al., 1990; Stoodley et al., 1997; Brodbelt et al., 2003). CSF and circulating blood have the potential to interact at any point within the intracranial space; thus, potential for regional cooling or warming at the capillary level can occur simultaneously within the brain parenchyma. A recent study of ventricular CSF temperature in normal pressure hydrocephalus (NPH) demonstrated increased periventricular cerebral temperatures in NPH which were corrected with shunting (Kuriyama et al., 2015) suggesting normal ventricular shape and size are important for cerebral temperature regulation. Through this extensive and intimate fluid-brain and fluid-vessel interactions, the biochemical and thermal environment of the CNS can be finely tuned and regulated.

## Meninges

Human meninges are regarded as connective tissue with a low metabolic rate; yet, they have an extraordinarily dense and robust vascular network with an intriguingly complex anatomy of arteries and veins (Kerber and Newton, 1973; Roland et al., 1987). The middle meningeal vessels are significantly more developed in *Homo sapiens* than other extinct human species based on fossilized remains and angiotomography (Bruner and Sherkat, 2008; Bruner et al., 2011). The meninges seem to possess the same autoregulatory ability as the cortical circulation to manage blood flow through intrinsic, non-neuronal mechanisms (Michalicek et al., 1996). Dural blood flow in healthy young adult humans has an average whole brain blood flow of approximately 46 ml · min^−1^ ·100 g^−1^ (Mchenry et al., 1978; Chen et al., 1984; Faraci et al., 1989). This remarkable physiological property has led to the development of various neurosurgical techniques to optimize the use of meninges for cortical revascularization (McLaughlin and Martin, 2013). Conversely, traumatic injury to the meningeal vasculature may prove fatal. Left untreated, traumatic injury to the meninges resulted in 4.8% mortality in one study of hospital admissions (Irie et al., 2011). The elaboration of an extraordinarily dense and robust meningeal vascular network with autoregulatory capacity implies a more important role for the meninges than merely a structural one. Adjacent to the CSF compartment, the human meninges may provide not only mechanical protection but also thermal regulation for the CNS (Zenker and Kubik, 1996).

## Skull

The skull consists of inner and outer tables of compact bone, and a central cancellous layer. The middle layer or diploë of the skull contains a network of numerous veins and venous lakes. Together with the transosseous emissary or perforating veins, diploic vessels represent an anastomotic system between the intracranial and extracranial venous systems. Devoid of valves, the diploic veins freely communicate with dural sinuses and pericranial veins and facilitate bi-directional blood flow and heat transfer interactions between the scalp and the brain surface. The skull thus serves not only as a bony structure, but also contributes to cranial thermal regulation. In addition, the modern human skull has an extremely globular shape (Bruner, 2004) that minimizes surface area to volume ratio and therefore further protects the brain from environmental thermal challenges.

## Scalp

The scalp has unique anatomical and physiological properties that are relevant to the thermal interactions between the brain and the external environment. For example, the forehead has one of the highest sweat gland densities and a greater sweat response, facilitating evaporative cooling of the brain during thermal loading (Cotter et al., 1995). The scalp blood vessels provide relatively high, constant blood flow compared with the rest of the body, with the ratio of scalp blood flow to surface area 4–10 times greater than that of the trunk and proximal limbs (Hertzman and Randall, 1948). Additionally, the scalp blood vessels demonstrate little or no vasoconstriction in response to cold (Froese and Burton, 1957), acting as a potential mechanism of enhanced evaporative cooling. The robust and relatively constant blood flow in the scalp provides a vascular thermal shielding mechanism to help maintain brain temperature homeostasis.

Conversely, although the head and neck represent only 7–9% of the total body surface area, even a small increase in heat loss from surface cooling of this region causes a relatively larger cooling of the body core (Pretorius et al., 2006). Clinically, because of the unique physiological characteristics, scalp arteries are routinely used as donor vessels for extracranial-intracranial bypass procedures to enhance cerebral vascularization; however these same properties give rise to the possibility that simple lacerations of the scalp may result in hemorrhagic shock or even prove fatal (Lemos and Clark, 1988). In sum, the scalp has features, including sweat glands and specialized blood vessels that promote uniquely rapid thermal homeostasis of the brain.

## Conclusion

Intricate temperature regulation co-evolved with increasing neural complexity, implying interdependence between temperature regulation and the achievement of higher functioning states. Temperature regulation of the brain is a dynamic parameter that is responsive to brain function and can even transfer heat from one brain region to another—essentially cooling and heating simultaneously. Brain temperature fluctuation studies can be traced back to the 1960s, where the effects of sleep, sensory stimulation, and environmental challenges affected regional brain temperature (reviewed in Wang et al., 2014). Learning and memory in awake and freely moving rats demonstrated increased hippocampal temperature in exploring rats, vs. a decrease in quiet resting animals suggesting function dependent changes in metabolism and local brain temperature. This spatiotemporally appropriate regulation involves blood circulating in the arterial and venous systems, CSF, and the separate but contiguous meningeal circulation, with the entire system responsive to both brain function and environment (Wang et al., 2014). The underlying mechanisms could be exploited with potentially great clinical effect in cases of traumatic or ischemic injury, high fever, encephalopathy or other conditions if those mechanisms were fully elucidated.

The calvaria and its components may function as a heat shield, or more accurately, a series of heat shields—with the bony skull as a conventional insulating heat shield and the meninges and scalp representing intra- and extracranial thermal soak heat shields with efficiencies dictated by blood flow. Furthermore, the scalp has the capacity to act as an ablative heat shield through the evaporation of sweat. The brain may rely principally on one or the other system, such as in warm weather or during fever; thus, a multifaceted and robust heat shielding system is formed.

The “central fever” associated with multiple types of cerebral insult including TBI, intracranial hemorrhage, and ischemic stroke is likely a compensatory measure which may help to overcome energetic barriers at the molecular and cellular levels, particularly at synapses, and may be similar to fever as a response to infection (reviewed in Wang et al., 2014). Just as in septic patients, more severe injury correlates to higher temperature and worse outcomes. Mitochondrial uncoupling proteins (UCPs), particularly UCP2, have been of particular interest with respect to electrochemical and thermal compensatory changes (as reviewed in Wang et al., 2014). Current understanding of UCP2 and its involvement in physiological post-injury electrical potential (ΔΨm) mitigation and associated heat generation indicates that cooling may provide a heat sink to increase the electrochemical rate of compensation, although increased heat may also result in greater synaptic activity which may make a higher temperature critical for compensation (Wang et al., 2014).

With the advancement of non-invasive imaging techniques, MR thermometry is a tool that has been used for brain thermal changes. Current uses for thermal guided procedures have been utilized primarily for thermal ablation for drug resistant epilepsy surgeries (McCracken et al., 2015), however MR thermometry for TBI or other types of cerebral insults have not yet been established, and continues to be an area for further investigation. Better understanding of cerebral heat management has great potential to guide augmented physiological responses to injury and insult without altering cerebral function to help improve patient outcomes. Perhaps if the regulatory mechanisms governing brain thermoregulation were better understood, it may be possible to locally cool targeted brain regions while allowing other regions to remain warm or even actively warm them simultaneously—just as the human cranial system does each day.

## Author contributions

HW brought the manuscript to fruition through the collection of historical and current trends in neuroanatomy and thermal regulation and composed early drafts of themanuscript. MK, KN contributed to each section including major revisions, current trends and research in each area, and facilitated with manuscript editing and figure assembly. DL provided critical feedback and manuscript revisions and significant intellectual commentary on the manuscript.

### Conflict of interest statement

The authors declare that the research was conducted in the absence of any commercial or financial relationships that could be construed as a potential conflict of interest.
